# JISP is in PubMed

**DOI:** 10.4103/0972-124X.65424

**Published:** 2010

**Authors:** D. Arunachalam

**Affiliations:** *Editor, Journal of Indian Society of Periodontology, H 11 A South Avenue, Thiruvanmiyur, Chennai - 600 041, India. E-Mail: editor@jisponline.com*

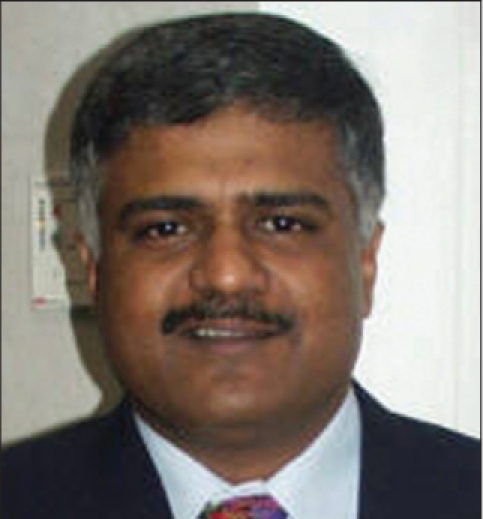



I am immensely pleased and happy to inform you that our Journal, the Journal of the Indian Society of Periodontology (JISP), is now indexed with PubMed. One will be able to search the articles published in the journal from 2008 onwards in PubMed via the following link: http://www.ncbi.nlm.nih.gov/sites/entrez?db=pubmed&cmd=DetailsSearch&term=J+Indian+Soc+Periodontol

It was in 2008 when JISP became synonymous with www.jisponline.com, a peer-reviewed journal. The online submission/revision of the articles using a unique ID through www.journalonweb.com/jisp increased accessibility, eased monitoring and processing, and at the same time eliminated any bias during peer-review process. The full text of the journal is also archived with PMC and searchable separately from Entrez PubMed.

The ISP is really fortunate to have a wealth of experienced academicians who have painstakingly reviewed and corrected the articles that the young minds bring in. I would like to particularly thank the publishers, Medknow Publications, and specifically Dr. D.K. Sahu, the Director for their sincere efforts, and mention my appreciation for the efforts put in by Dr. Neha Gupta for sharing the manuscript and copy editing with me.

Our next goal is indexing JISP with Science Citation Index which seems impossible without your comments, critique and suggestions.

As this being one of the most important milestones, it is important that we continue to maintain and improve the quality of papers by rigorous peer-review and maintain the publication schedule so as to take the maximum advantage of PubMed indexing.

I look forward to your continuing support as always.

